# Usefulness of Preradioactive Iodine Scans in Thyroid Cancer

**DOI:** 10.1210/jendso/bvaf128

**Published:** 2025-08-04

**Authors:** Gayatri Jaiswal, Michael Grimes, Patricia Bononi, Nishit Vaghasia, Saira Khan, Kersthine Andre, Ashni Dharia, Jamil Alkhaddo

**Affiliations:** Center for Diabetes and Endocrine Health, Allegheny Health Network, Medicine Institute, Pittsburgh, PA 15017, USA; Center for Diabetes and Endocrine Health, Allegheny Health Network, Medicine Institute, Pittsburgh, PA 15017, USA; Center for Diabetes and Endocrine Health, Allegheny Health Network, Medicine Institute, Pittsburgh, PA 15017, USA; Center for Diabetes and Endocrine Health, Allegheny Health Network, Medicine Institute, Pittsburgh, PA 15017, USA; Center for Diabetes and Endocrine Health, Allegheny Health Network, Medicine Institute, Pittsburgh, PA 15017, USA; Center for Diabetes and Endocrine Health, Allegheny Health Network, Medicine Institute, Pittsburgh, PA 15017, USA; Division of Internal Medicine, Allegheny Health Network, Medicine Institute, Pittsburgh, PA 15212, USA; Center for Diabetes and Endocrine Health, Allegheny Health Network, Medicine Institute, Pittsburgh, PA 15017, USA

**Keywords:** radioactive iodine, thyroid cancer, whole body iodine scan

## Abstract

**Context:**

There is an evolving role for radioactive iodine (RAI) in thyroid cancer treatment. Radioactive iodine treatment usually involves a pre-RAI whole-body iodine scan and a posttherapy scan. The clinical utility of pre-RAI therapy scans has been increasingly questioned.

**Aim:**

To evaluate the clinical utility of pre-RAI whole-body iodine scans.

**Methods:**

We retrospectively reviewed the medical records of differentiated thyroid cancer patients treated with RAI. Using records blinded for pre-RAI scans, 3 endocrinologists developed empiric RAI treatment plans for each patient based on surgical pathology. This was repeated using the unblinded records, and the treatment plans made with and without pre-RAI scan results were compared.

**Results:**

A total of 164 patients met the inclusion criteria: 89 patients (54.3%) were low risk, 61 (37.2%) intermediate risk, and 14 (8.5%) high risk for thyroid cancer recurrence. After blinded review, RAI treatment was recommended for 122 patients (74.3%); 46 were determined to be appropriate for a low-dose RAI, 75 a medium dose, and 1 a high dose. When unblinded, different recommendations were made for only 7 patients (5.7%), with 6 being recommended for a higher RAI dose. In addition, the prescan RAI results prompted recommendations for additional testing, such as neck ultrasounds or computed tomography or postoperative thyroglobulin levels. Pre-RAI scans affected patient care plans in only 7 (5.7%) of the 164 patients in the study.

**Conclusion:**

In most patients with thyroid cancer who may need RAI, pre-RAI scans may not affect management, and empiric RAI doses may be a more cost-effective and convenient option.

Thyroid cancer accounts for approximately 2.2% of all new cancer cases and affected an estimated 44 020 people in the United States in 2024 [1]. Recent advances in thyroid cancer management have enabled a more individualized approach to treatment, with an evolving role for radioactive iodine (RAI). Radioactive iodine treatment usually involves a pre-RAI whole-body iodine scan and a posttherapy scan. The role of planar pretherapy diagnostic RAI scans (pre-RAI scan) in patients undergoing RAI therapy is controversial. Posttreatment scans are recommended for staging RAI-avid disease and identifying RAI-refractory tumors, per a joint statement from the American Thyroid Association (ATA), the European Association of Nuclear Medicine, the European Thyroid Association, and the Society of Nuclear Medicine and Molecular Imaging [2]. However, the joint statement also suggests that the utility of pretreatment diagnostic RAI molecular imaging warrants reevaluation [2]. Here, we evaluated the clinical utility of pre-RAI scans to assess their value in contemporary thyroid cancer management.

RAI therapy has been used to diagnose and manage patients with well-differentiated thyroid cancer since the 1940s [3]. During RAI therapy, iodine 131 (^131^I) is taken up by thyroid tissue and concentrated in thyroid follicular cells, causing thyroid cell death via emitted β particles [4]. Iodine 131 also emits γ radiation, which is used for scanning and visualizing the uptake [4]. In contrast, ^123^I is primarily a γ emitter that does not impair cellular function, produces superior images, and is more expensive than ^131^I [4]. Both ^131^I and ^123^I can be used for a diagnostic, whole-body scan (pre-RAI scan) to identify local, regional, and distant metastases. The results of these pre-RAI scans are intended to inform RAI treatment decisions, including dose modification, and these decisions can potentially impact disease-free survival and overall mortality. The role of pre-RAI scans in deciding to administer RAI is increasingly questioned. Concerns also exist regarding pre-RAI scans with ^131^I potentially reducing RAI therapy efficacy because of a “stunning” effect [4, 5]. An alternative treatment strategy involves empiric RAI ablation without a pre-RAI scan. The need for pre-RAI scans has been reduced by the development of several new tools, including high-quality neck ultrasounds, postoperative thyroglobulin levels as a prognostic measure, and the emergence of molecular markers/next-generation sequencing for risk stratification. When pre-RAI scans are used, they can help localize regional and distant metastatic disease [6]. The pre-RAI scan is also useful for evaluating the capacity of metastatic deposits to concentrate ^131^I [6].

Different institutions have variable protocols regarding scanning prior to and after RAI treatment. Some use ^123^I planar scans or ^131^I single-photon emission computed tomography (CT)-CT scans for all patients who are potential candidates for RAI treatment. The pre-RAI scan results may provide guidance for additional surgery or alter the prescribed ^131^I therapy. The ^131^I therapy may be altered by adjusting empiric ^131^I activity or recalculating dosimetry to the maximum tolerated therapeutic ^131^I activity for treatment of distant metastatic disease [7]. After thyroid surgery, most patients have a thyroid remnant, and some centers use empiric RAI therapy (decided based on initial risk stratification) with a post-RAI ^131^I scan.

In our large health network, planar ^123^I pre-RAI therapy scans are used as diagnostic scans for all patients. Whether patients are candidates for RAI treatment is decided by their treating endocrinologist. The RAI dose is often a shared decision by the endocrinologist and the nuclear medicine radiologist. Clear guidelines have not yet been developed for pre-RAI scans in patients perceived to have low- to intermediate-risk thyroid cancer [2].

We assessed the clinical utility of ^123^I scans before RAI treatment in thyroid cancer patients by comparing the management decisions of endocrinologists before and after reviewing patient pre-RAI scan results. The intention of the study was to evaluate the impact of pre-RAI scans on dosing decisions related to ^131^I therapy.

## Methods

This quality improvement project was granted institutional review board exemption by the Allegheny Health Network institutional review board.

We retrospectively reviewed the medical charts of patients with differentiated thyroid cancer who received RAI therapy (initial treatment) at the Nuclear Medicine Department at Allegheny General Hospital from January 1, 2016, through December 31, 2021. Patients were referred to RAI therapy by their endocrinologist based on individual risk assessment. We included differentiated thyroid cancer patients aged ≥ 18 years who received a total or near total thyroidectomy with thyroid hormone withdrawal or thyrotropin alfa injections. We excluded patients without pre-RAI scans (whole-body iodine scans), post-RAI whole-body scans (post-RAI scans), or data regarding their initial pathology.

RAI treatment and dosage decisions were made at the treating endocrinologist and nuclear medicine physician's discretion. During the 2 weeks before treatment, patients maintained a low-iodine diet. The imaging protocol included a pretreatment ^123^I whole-body planar scan following thyrotropin alfa stimulation or thyroid hormone withdrawal. This scan was performed 1 day before RAI administration with ^131^I and followed by a posttreatment ^131^I scan 7 days later.

For this study, clinical, surgical pathology, and imaging data were collected for each patient. Using these clinical and surgical data, 3 endocrinologists determined RAI treatment indication and dose for each patient while blinded to pre-RAI and post-RAI whole-body scan results and RAI treatment details. Each endocrinologist performed initial risk stratification for each patient based on histopathology information and classified the patients as low, intermediate, or high risk for recurrence according to ATA guidelines [2, 8, 9]. Low-risk patients had no local or distant metastases, complete macroscopic tumor resection, no evidence of locoregional tumor invasion, and nonaggressive tumor histology. For intermediate-risk patients, there was histopathological evidence of microscopic invasion of tumor into perithyroidal soft tissues, cervical lymph node metastases in the surgical specimens or tumors with aggressive histology, or minimal vascular invasion. High-risk patients had histopathological evidence of macroscopic tumor invasion, incomplete tumor resection, or clinical information disclosing distant metastases.

Using the initial risk stratification, the endocrinologists made the decision to withhold or to administer RAI and determined the prescribed ^131^I activity: low 30 to 50 mCi, medium 75 to 100 mCi, or high 150 to 200 dose ^131^I therapy. Treatment recommendations were based on the 2015 ATA thyroid cancer guidelines [9]. After unblinding the pre-RAI scans, the endocrinologists reanalyzed the patients' data to establish if there was any management change that they would make based on the additional information they had from these scans.

### Statistical Analysis

Categorical variables were tabulated as counts with calculated percentages. Continuous variables were evaluated using a mean ± SD, mean (95% CI), or median (25th; 75th percentile), depending on the distribution.

## Results

We reviewed 195 charts of patients with differentiated thyroid cancer, but only 164 patients had complete data and were included in the final analysis. The study population consisted of 104 (63%) female patients and 60 (37%) male patients, and the median age was 51 years (Table 1). The mean tumor size was 2.4 cm, with a median size of 1.8 cm. Complete patient and tumor characteristics data are summarized in Table 1, including age, gender, tumor size, subtype, histopathology, laterality, focality, vascular involvement and extrathyroidal extension, surgical margins, and neck node metastasis.

**Table 1.** Patient demographics and tumor history for the 164 patients meeting criteria for study inclusion

	Median (range), n (%)
Age (y)	51 (21-90)
	**n (%)**
Age (y)	
<45	64 (39)
≥45	100 (61)
Gender	
Female	104 (63.4)
Male	60 (36.6)
Tumor subtypes	
Papillary	152 (92.7)
Follicular	11 (6.7)
Hurthle cell variant	1 (0.6)
Subtype of papillary thyroid cancer	
Papillary classic	94 (61.8)
Follicular variant	38 (25)
Classic plus follicular variant	12 (7.9)
Tall cell + classic	2 (1.3)
Columnar	0
Cystic	0
Sclerosing	1 (0.6)
Squamous	0
Insular	0
Classic + oncocytic	1 (0.6)
Tall cell + oncocytic	1 (0.6)
Not classified	3 (2.0)
**Histology**	
Mean tumor size	2.4 cm
Median tumor size	1.8 cm
Tumor laterality	
Right	66 (40.2)
Left	41 (25.0)
Bilateral	57 (34.8)
Multifocal	85 (51.8)
Vascular invasion	24 (14.6)
Extra-Thyroidal extension	31 (20.4)
Surgical margins	
Positive	34 (20.7)
Negative	130 (79.3)
Neck node metastasis	
Positive	55 (33.5)
Negative	65 (39.6)
Unknown	44 (26.8)

Using the initial pathology results and ATA risk category criteria [9, 10], endocrinologists determined histopathological staging and ATA risk stratification categories (Tables 2 and 3). Eighty-nine of 164 patients (54.3%) were classified as low risk, 61 of 164 patients (37.2%) were intermediate risk, and 14 of 164 patients (8.5%) were high risk. The reviewing endocrinologists thought RAI treatment was not indicated in 42 patients (25.6%) and was recommended for 122 patients (74.3%) [9]. Of those recommended for treatment, 46 patients were determined to be appropriate for a low-dose RAI, 75 to receive a medium dose and 1 patient to receive a high dose.

**Table 2.** Impact of final risk stratification on the prescribed iodine 131 (^131^I) dose for 164 patients when evaluated blindly

		Prescribed ^131^I activity			
Risk	Patients, n (%)	None	30-50 mCi	75-100 mCi	150-200 mCi
Low	89 (54.3)	42	46	1	0
Intermediate	61 (37.2)	0	0	60	1
High	14 (8.5)	0	0	1	13

**Table 3.** Histopathology of tumor (T) components and nodal (N) status as classified for risk by the endocrinologist according to the risk stratification guidelines of the American Thyroid Association

	Patients	Low risk	Intermediate risk	High risk
**Tumor**	**n (%)**	**n (%)**	**n (%)**	**n (%)**
T1a ≤ 1.0 cm	19 (11.6)	16 (9.8)	2 (1.2)	1 (0.6)
T1b 1.0 cm to 2.0 cm	55 (33.5)	41 (25.0)	9 (5.5)	5 (3.0)
T2 > 2.0 cm to 4.0 cm	44 (26.8)	30 (18.3)	14 (8.5)	0
T3 > 4.0 cm or mETE	43 (26.2)	2 (1.2)	36 (22.0)	5 (3.0)
T4	3 (1.8)	0	0	3 (1.8)
Total	164 (100)	89 (54.3)	61 (37.2)	14 (8.5)
**Node**				
N0 benign lymph nodes	65 (39.6)	38 (23.2)	24 (14.6)	3 (1.8)
N1a metastatic level VI nodes*^a^*	29 (17.7)	13 (7.9)	16 (9.8)	0
N1b metastatic levels I-V, VII nodes	26 (15.9)	6 (3.7)	13 (7.9)	7 (4.6)
No surgical nodal specimen	44 (26.8)	32 (19.5)	8 (4.9)	4 (2.4)
Total	164 (100)	89 (54.3)	61 (37.2)	14 (8.5)

Abbreviation: mETE, minimal extrathyroidal extension.

^a^Node metastatic levels defined as level VI: central neck compartment, levels I-V: lateral neck compartment, level VII: superior mediastinum.

When reviewing the unblinded diagnostic ^123^I scans, the endocrinologists identified 20 of 164 patients (12.2%) with possible regional metastases, 6 of 164 patients (3.7%) with likely regional metastases, and 1 of 164 patients (0.6%) with distant metastases. The information obtained from the diagnostic ^123^I scans led to changes in RAI dose for 7 of 164 patients (4.2%). Among these, 3 were classified as intermediate risk, 3 as high risk, and 1 as low risk (Table 4). In 6 of 7 cases with different management decisions, the pre-RAI scan suggested a higher RAI dose than initially warranted to effectively target the detected residual or metastatic disease. The increase was typically from a standard ablative dose (30-50 mCi) to a higher dose (75-100 mCi). Across all 7 cases, the pre-RAI scan findings prompted the reviewing endocrinologists to recommend additional imaging, such as a neck ultrasound or CT scan, to better characterize the extent of the disease and guide treatment decisions (Table 4).

**Table 4.** Summary of tumor information and prescribed treatments in the 7 patients with different care plans before and after unblinding

Age (y)	Tumor type	Tumor size (cm)	Initial staging	Pre-RAI findings	Recommended action
56	PTC	1.2	T1bN1bM0	Uptake in thyroid bed, lateral neck, and right para midline neck (indeterminate for remnant or metastasis)	Higher RAI dose (75-100 mCi), possibly neck ultrasound
60	PTC (follicular and solid variants)	4.1	T3N0M0	Substantial residual thyroid tissue, three foci suspicious for contralateral lymph node metastases	Higher RAI dose (75-100 mCi), neck ultrasound, thyroglobulin levels
61	PTC (Tall cell, oncocytic features)	1.5	T4NxMx	Uptake in right para midline low neck and three irregular foci in right lateral neck (likely metastatic lymph nodes)	Further imaging (lymph node dissection), RAI dose may not change due to high-risk pathology
25	PTC	3.0	T2NxM0	Intense uptake in thyroid bed and inferior focus (residual/metastatic)	Further imaging (CT neck or ultrasound), potentially higher RAI dose
54	OTC	4.4	T3N0M0	Significant uptake in thyroid bed and lateral neck (bilateral)	Further imaging (CT neck or ultrasound)
71	PTC	1.2	T1bNxM0	Thyroid bed uptake and upper mediastinum uptake	Potentially higher RAI dose, further imaging
66	PTC multifocal	2.4	T2N0M0	Moderate uptake in thyroid bed and left side (possible lymph node metastasis)	Potentially higher RAI dose, neck ultrasound or CT scan

Abbreviations: OTC, oncocytic thyroid cancer; PTC, papillary thyroid cancer; RAI, radioactive iodine.

## Discussion

Our results demonstrate that if the RAI dose was determined purely based on clinical and laboratory/pathology data in the absence of pre-RAI scans, management would not have changed in the majority (95%) of the cases. In patients who would be candidates for RAI based on 2015 ATA guidelines, only 7/122 (5.7%) patients may have had their treatment changed based on pretreatment ^123^I scans. Reducing pre-RAI scans may reduce health care burdens and treatment costs while not compromising overall outcomes.

Although the ATA advocates for posttreatment scans to be performed, there are no clear guidelines on when to perform pre-RAI scans in patients who would receive RAI therapy [2]. Thyroid cancer management has become more individualized since the ATA guidelines were released in 2015, with RAI being used less frequently for remnant ablation of low-risk thyroid cancers [11, 12]. Other published statements from multiple medical associations recommend the provider consider pre-RAI scans in the context of their patient's surgery status and other individualized factors [2, 4, 13].

The decision to conduct a pre-RAI scan may influence the overall ^131^I activity a patient receives over the course of their treatment. Pre-RAI scans have a low theragnostic power due to radioiodine indifference and low detection sensitivity for small-volume cervical nodal disease. Preablation whole-body imaging cannot differentiate between cervical metastatic residual disease and benign thyroid remnants [14]. Over- and undertreatment are significant concerns for patients with tumors that have low RAI uptake in a pretreatment scan, or in cases where there is no differentiation between thyroid remnants and lymph node involvement [14]. Newer tools such as thyroglobulin level assays and high-quality ultrasounds are available before RAI treatment and are vital in developing individualized treatment plans that account for pre-RAI scan limitations. In addition, metastasis can often be predicted based on these assays.

Bravo et al [14] showed 30% discordance between pre-RAI scans vs post-RAI scans. Their study indicated the post-RAI scan appears to yield greater iodine-avid disease detection than the pre-RAI study in patients with clinical evidence of persistent disease after RAI and/or distant metastasis (M1 status) [14]. This raises the question of the role of these pre-RAI planning scans in this clinical scenario. Using pre-RAI scans is especially relevant in patients with suspected or known M1 because in this cohort, pre-RAI scans are often used in the decision-making for delivery of higher RAI doses.

In the current study, there were 8 patients where some of the findings seen on the pre-RAI scan would have been found on the posttreatment scan and led to further neck imaging, etc. However, in 7 (87.5%) of these patients, the RAI dose would have changed based on the pre-RAI scan. It is uncertain whether this change would have made a long-term difference in disease-free survival or overall mortality. Pathology findings placed most of these patients in the intermediate- or high-risk categories, and it is uncertain if postoperative thyroglobulin levels or perioperative imaging (high-quality neck ultrasound or CT scans) would give us comparative information to a pre-RAI scan. This is in line Santhanam et al [15], who concluded pretherapy scans may not have an effect on the total dose of ^131^I a patient receives, especially in individuals found to have low-risk tumors.

Although we did not evaluate using pre-RAI scans in the context of cost-effectiveness, there are potential cost savings in limiting pre-RAI scans to high-risk cases. In our own study, prescan data affected the prescribed treatments of only 7 patients (5.7%). This is in line with the findings of van der Boom et al [16], who showed that pre-RAI changed management in only 4% of patients receiving RAI. Arjani et al [17] used a decision tree model to evaluate cost-effectiveness of pre-RAI scans and concluded it was more cost-effective to administer RAI empirically. In contrast, Van Nostrand et al [18] showed that ablation RAI scans results may have changed the RAI treatments in 53% of the patients evaluated in their retrospective study. Many of the studies discussed here, including our own, use retrospective approaches.

Limitations

Our study has some limitations. First, it is retrospective in nature, which can introduce selection bias. Second, the decisions regarding risk stratification and interpretation of pre-RAI scan reports were subjective interpretations by the 3 reviewing endocrinologists. Although these endocrinologists are experienced in thyroid cancer management, these results may not be reproducible with a different group of endocrinologists. Third, we did not include thyroglobulin levels or neck ultrasound results in the patient information reviewed by the endocrinologists, which could further help in risk stratification and treatment decisions. Fourth, some of the patients who were recommended treatment would not have received RAI in other hospitals and clinics, especially in the current clinical era, as most of these patients have a low risk of recurrence. This may influence the population in the study. Finally, our findings describe the experience acquired with planar ^123^I scans and cannot be generalized to centers using nuclear images with single-photon emission CT-CT scans.

## Conclusion

In our retrospective chart review of patients with differentiated thyroid cancer, pre-RAI scans before RAI treatment may have changed management in only 5.7% of cases, especially in the low- to intermediate-risk patients. Large-scale prospective studies are needed to develop clear guidelines for treating thyroid cancer patients, including intermediate or high-risk patients. Such studies may not only improve patient outcomes and minimize their long-term risks, but also potentially lead to cost savings.

## Data Availability

Some or all datasets generated during and/or analyzed during the current study are not publicly available but are available from the corresponding author on reasonable request.

## Abbreviations

ATA: American Thyroid Association
CT: computed tomography
RAI: radioactive iodine
^131^I: iodine 131
